# A topological model for partial equivariance in deep learning and data analysis

**DOI:** 10.3389/frai.2023.1272619

**Published:** 2023-12-21

**Authors:** Lucia Ferrari, Patrizio Frosini, Nicola Quercioli, Francesca Tombari

**Affiliations:** ^1^Department of Mathematics, University of Bologna, Bologna, Italy; ^2^Department of Electrical, Electronic, and Information Engineering (DEI) and WiLab-National Laboratory for Wireless Communications, National Inter-University Consortium for Telecommunications (CNIT), University of Bologna, Bologna, Italy; ^3^Department of Mathematics, Royal Institute of Technology (KTH), Stockholm, Sweden

**Keywords:** partial-equivariant neural network, P-GENEO, pseudo-metric space, compactness, convexity

## Abstract

In this article, we propose a topological model to encode partial equivariance in neural networks. To this end, we introduce a class of operators, called P-GENEOs, that change data expressed by measurements, respecting the action of certain sets of transformations, in a non-expansive way. If the set of transformations acting is a group, we obtain the so-called GENEOs. We then study the spaces of measurements, whose domains are subjected to the action of certain self-maps and the space of P-GENEOs between these spaces. We define pseudo-metrics on them and show some properties of the resulting spaces. In particular, we show how such spaces have convenient approximation and convexity properties.

## 1 Introduction

**Figure 1:**
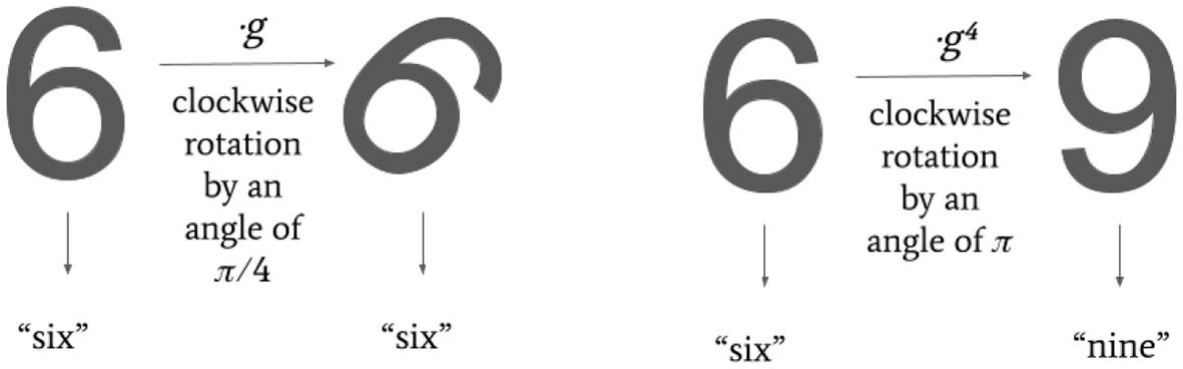
Example of a symmetry breaking feature. Applying a rotation *g* of π/4, the digit “6” preserves its meaning **(left)**. The rotation *g*^4^ of π is, instead, not admissible, since it transforms the digit “6” into the digit “9” **(right)**.

Over the past decade, several geometric techniques have been incorporated into Deep Learning (DL), giving rise to the new field of Geometric Deep Learning (GDL) (Cohen and Welling, 2016; Masci et al., 2016; Bronstein et al., 2017). This geometric approach to deep learning is exploited with a dual purpose. On one hand, geometry provides a common mathematical framework to study neural network architectures. On the other hand, a geometric bias, based on prior knowledge of the data set, can be incorporated into DL models. In this second case, GDL models take advantage of the symmetries imposed by an observer, which encode and elaborate the data. The general blueprint of many deep learning architectures is modeled by group equivariance to encode such properties. If we consider measurements on a data set and a group encoding their symmetries, i.e., transformations taking admissible measurements (for example, rotation or translation of an image), the group equivariance is the property guaranteeing that such symmetries are preserved after applying an operator (e.g., a layer in a neural network) on the observed data. In particular, let us assume that the input measurements Φ, the output measurements Ψ and, respectively, their symmetry groups *G* and *H* are given. Then the agent *F*: Φ → Ψ is *T*-equivariant if *F*(*φg*) = *F*(*φ*)*T*(*g*), for any *φ* in Φ and any *g* in *G*, where *T* is a group homomorphism from *G* to *H*. In the theory of Group Equivariant Non-Expansive Operators (GENEOs) (Camporesi et al., 2018; Bergomi et al., 2019; Cascarano et al., 2021; Bocchi et al., 2022, 2023; Conti et al., 2022; Frosini et al., 2023; Micheletti, 2023), as in many other GDL models, the collection of all symmetries is represented by a group, but in some applications, the group axioms do not necessarily hold since real-world data rarely follow strict mathematical symmetries due to noise, incompleteness, or symmetry-breaking features. As an example, we can consider a data set that contains images of digits and the group of rotations as the group acting on it. Rotating an image of the digit “6” by a straight angle returns an image that the user would most likely interpret as “9”. At the same time, we may want to be able to rotate the digit “6” by small angles while preserving its meaning (see Figure 1).

It is then desirable to extend the theory of GENEOs by relaxing the hypotheses on the sets of transformations. The main aim of this article is to give a generalization of the results obtained for GENEOs to a new mathematical framework, where the property of equivariance is maintained only for some transformations of the measurements, encoding a partial equivariance with respect to the action of the group of all transformations. To this end, we introduce the concept of Partial Group Equivariant Non-Expansive Operator (P-GENEO).

In this new model, there are some substantial differences with respect to the theory of GENEOs:

1. The user chooses two sets of measurements in input: the one containing the original measurements and another set that encloses the admissible variations of such measurements, defined in the same domain. For example, in the case where the function that represents the digit “6” is being observed, we define an initial space that contains this function and another space that contains certain small rotations of “6” but excludes all the others.
2. Instead of considering a group of transformations, we consider a set containing only those that do not change the meaning of our data, i.e., only those associating with each original measurement another one inside the set of its admissible variations. Therefore, by choosing the initial spaces, the user defines also which transformations of the data set, given by right composition, are admissible and which ones are not.
3. We define partial GENEOs, or P-GENEOs, as a generalization of GENEOs. P-GENEOs are operators that respect the two sets of measurements in input and the set of transformations relating them. The term “partial” refers to the fact that the set of transformations does not necessarily need to be a group.

With these assumptions in mind, we will extend the results proven in the study by Bergomi et al. (2019) and Quercioli (2021a) for GENEOs. We will define suitable pseudo-metrics on the spaces of measurements, the set of transformations, and the set of non-expansive operators. Grounding on their induced topological structures, we prove compactness and convexity of the space of P-GENEOs under the assumption that the function spaces are compact and convex. These are useful properties from a computational point of view. For example, compactness guarantees that the space can be approximated by a finite set. Moreover, convexity allows us to take the convex combination of P-GENEOs in order to generate new ones.

## 2 Related work

The main motivation for our study is that observed data rarely follow strict mathematical symmetries. This may be due, for example, to the presence of noise in data measurements. The idea of relaxing the hypothesis of equivariance in GDL and data analysis is not novel, as it is shown by the recent increase in the number of publications in this area (see, for example, Weiler and Cesa, 2019; Finzi et al., 2021; Romero and Lohit, 2022; van der Ouderaa et al., 2022; Wang et al., 2022; Chachlski et al., 2023).

We identify two main ways to transform data *via* operators that are not strictly equivariant due to the lack of strict symmetries of the measurements. On one hand, one could define *approximately equivariant* operator. These are operators for which equivariance holds up to small perturbation. In this case, given two groups, *G* and *H* acting on the spaces of measurements Φ and Ψ, respectively, and a homomorphism between them, *T*: *G* → *H*, we say that *F*: Φ → Ψ is *ε*-equivariant if, for any *g* ∈ *G* and *φ* ∈ Φ, ||*F*(*φg*) − *F*(*φ*)*T*(*g*)||_∞_ ≤ *ε*. Alternatively, when defining operators transforming the measurements of certain data sets, equivariance may be substituted by *partial equivariance*. In this case, equivariance is guaranteed for a subset of the groups acting on the space of measurements, with no guarantees for this subset to be a subgroup. Among the previously cited articles about relaxing the property of equivariance in DL, the approach by Finzi et al. (2021) is closer to an approximate equivariance model. Here, the authors use a Bayesian approach to introduce an inductive bias in their network that is sensitive to approximate symmetry. The authors of Romero and Lohit (2022) utilize a partial equivariance approach, where a probability distribution is defined and associated with each group convolutional layer of the architecture, and the parameters defining it are either learnt, to achieve equivariance, or partially learnt, to achieve partial equivariance. The importance of choosing equivariance with respect to different acting groups on each layer of the CNN was actually first observed in the study by Weiler and Cesa (2019) for the group of Euclidean isometries in ℝ^2^.

The point of view of this article is closer to the latter. Our P-GENEOs are indeed operators that preserve the action of certain sets ruling the admissibility of the transformations of the measurements of our data sets. Moreover, non-expansiveness plays a crucial role in our model. This is, in fact, the feature allowing us to obtain compactness and approximability in the space of operators, distinguishing our model from the existing literature on equivariant machine learning.

## 3 Mathematical setting

### 3.1 Data sets and operations

Consider a set *X* and the normed vector space $\left({\mathbb{R}}_{b}^{X},∥\cdot {∥}_{\infty }\right)$, where ${\mathbb{R}}_{b}^{X}$ is the space of all bounded real-valued functions on *X* and ∥·∥_∞_ is the usual uniform norm, i.e., for any $f\in {\mathbb{R}}_{b}^{X}$, $∥f{∥}_{\infty }:=\sup_{x\in X}|f(x)|$. On the set *X*, the space of transformations is given by elements of Aut(*X*), i.e., the group of bijections from *X* to itself. Then, we can consider the right group action R defined as follows (we represent composition as a juxtaposition of functions):


$$R:{\mathbb{R}}_{b}^{X}\times \text{Aut}\left(X\right)\to {\mathbb{R}}_{b}^{X},\;\left(\varphi ,s\right)\mapsto \varphi s.$$

*Remark 3.1.* For every *s* ∈ Aut(*X*), the map $R_{s}:{\mathbb{R}}_{b}^{X}\to {\mathbb{R}}_{b}^{X},$ with $R_{s}\left(\varphi \right):=\varphi s$ preserves the distances. In fact, for any $\varphi_{1},\varphi_{2}\in {\mathbb{R}}_{b}^{X}$, by bijectivity of *s*, we have that


$$\begin{array}{l} ∥R_{s}\left(\varphi_{1}\right)-R_{s}\left(\varphi_{2}\right){∥}_{\infty }=\underset{x\in X}{\sup}|\varphi_{1}s\left(x\right)-\varphi_{2}s\left(x\right)|\\ \;=\underset{y\in X}{\sup}|\varphi_{1}\left(y\right)-\varphi_{2}\left(y\right)|\\ \;=∥\varphi_{1}-\varphi_{2}{∥}_{\infty }. \end{array}$$

In our model, our data sets are represented as two sets Φ and Φ′ of bounded real-valued measurements on *X*. In particular, *X* represents the space where the measurements can be made, Φ is the space of permissible measurements, and Φ′ is a space which Φ can be transformed into, without changing the interpretation of its measurements after a transformation is applied. In other words, we want to be able to apply some admissible transformations on the space *X* so that the resulting changes in the measurements in Φ are contained in the space Φ′. Thus, in our model, we consider operations on *X* in the following way:

**Definition 3.2.** A (Φ, Φ′)**-operation** is an element *s* of Aut(*X*) such that, for any measurement *φ* ∈ Φ, the composition *φs* belongs to Φ′. The set of all (Φ, Φ′) operations is denoted by ${\text{Aut}}_{\Phi ,\Phi^{\prime }}\left(X\right)$.

*Remark 3.3*. We can observe that the identity function id_*X*_ is an element of ${\text{Aut}}_{\Phi ,\Phi^{\prime }}\left(X\right)$ if Φ ⊆ Φ′.

For any $s\in {\text{Aut}}_{\Phi ,\Phi^{\prime }}\left(X\right)$, the restriction to $\Phi \times \text{Au}{\text{t}}_{\Phi ,\Phi^{\prime }}\left(X\right)$ of the map $R_{s}$ takes values in Φ′ since $R_{s}\left(\varphi \right):=\varphi s\in \Phi^{\prime }$ for any *φ* ∈ Φ. We can consider the restriction of the map R (for simplicity, we will continue to use the same symbol to denote this restriction):


$$R:\Phi \times \text{Au}{\text{t}}_{\Phi ,\Phi^{\prime }}\left(X\right)\to \Phi^{\prime },\;\left(\varphi ,s\right)\mapsto \varphi s$$

where $R\left(\varphi ,s\right)=R_{s}\left(\varphi \right)$, for every $s\in {\text{Aut}}_{\Phi ,\Phi^{\prime }}\left(X\right)$ and every *φ* ∈ Φ.

**Definition 3.4.** Let *X* be a set. A **perception triple** is a triple (Φ, Φ′, *S*) with $\Phi ,\Phi^{\prime }\subseteq {\mathbb{R}}_{b}^{X}$ and $S\subseteq {\text{Aut}}_{\Phi ,\Phi^{\prime }}\left(X\right)$. The set *X* is called the **domain** of the perception triple and is denoted by dom(Φ, Φ′, *S*).

*Example 3.5.* Given *X* = ℝ^2^, consider two rectangles *R* and *R*′ in *X*. Assume Φ: = {*φ*: *X* → [0, 1]: supp(*φ*) ⊆ *R*} and Φ′: = {*φ*′:*X* → [0, 1]: supp(*φ*′) ⊆ *R*′}. We recall that, if we consider a function *f*: *X* → ℝ, the *support* of *f* is the set of points in the domain, where the function does not vanish, i.e., supp(*f*) = {*x* ∈ *X* | *f*(*x*) ≠ 0}. Consider *S* as the set of translations that bring *R* into *R*′. The triple (Φ, Φ′, *S*) is a perception triple. If Φ represents a set of gray level images, *S* determines which translations can be applied to our pictures.

### 3.2 Pseudo-metrics on data sets

In our model, considering a generic set *X*, data are represented by a space $\Omega \subseteq {\mathbb{R}}_{b}^{X}$ of bounded real-valued functions. We endow the real line ℝ with the usual Euclidean metric and the space *X* with an extended pseudo-metric induced by Ω:


$$D_{X}^{\Omega }\left(x_{1},x_{2}\right)=\underset{\omega \in \Omega }{\sup}|\omega \left(x_{1}\right)-\omega \left(x_{2}\right)|$$

for every *x*_1_, *x*_2_ ∈ *X*. The choice of this pseudo-metric over X means that two points can only be distinguished if they assume different values for some measurements. For example, if Φ contains only a constant function and *X* contains at least two points, the distance between any two points of *X* is always null.

The pseudo-metric space $X_{\Omega }:=\left(X,D_{X}^{\Omega }\right)$ can be considered as a topological space with the basis


$$B_{\Omega }={\left\{B_{\Omega }\left(x_{0},r\right)\right\}}_{x_{0}\in X,\;r\in {\mathbb{R}}^{+}}=\{\left\{x\in X:\;D_{X}^{\Omega }\left(x,x_{0}\right)<r\right\}{\}}_{x_{0}\in X,\;r\in {\mathbb{R}}^{+}},$$

and the induced topology is denoted by τ_Ω_. The reason for considering a topological space *X*, rather than just a set, follows from the need of formalizing the assumption that data are stable under small perturbations.

*Remark 3.6*. In our case, there are two collections of functions Φ and Φ′ in ${\mathbb{R}}_{b}^{X}$ representing our data, both of which induce a topology on *X*. Hence, in the model, we consider two pseudo-metric spaces *X*_Φ_ and $X_{\Phi^{\prime }}$ with the same underlying set *X*. If $\Phi \subseteq \Phi^{\prime }\subseteq {\mathbb{R}}_{b}^{X}$, the topologies τ_Φ_ and $\tau_{\Phi^{\prime }}$ are comparable and, in particular, $\tau_{\Phi^{\prime }}$ is finer than τ_Φ_.

Now, given a set $\Omega \subseteq {\mathbb{R}}_{b}^{X}$, we will prove a result about the compactness of the pseudo-metric space *X*_Ω_. Before proceeding, let us recall the following lemma (e.g., see Gaal, 1964):

**Lemma 3.7**. *Let (P,d) be a pseudo-metric space. The following conditions are equivalent*:

1. *P is totally bounded;*
2. *Every sequence in P admits a Cauchy subsequence.*

**Theorem 3.8**. *If* Ω *is totally bounded,* *X*_Ω_ *is totally bounded.*

Proof: By Lemma 3.7, it will suffice to prove that every sequence in *X* admits a Cauchy subsequence with respect to the pseudo-metric $D_{X}^{\Omega }$. A sequence (*x*_*i*_)_*i*∈ℕ_ in *X*_Ω_ is considered and a real number *ε* > 0 is taken. Since Ω is totally bounded, we can find a finite subset Ω_*ε*_ = {ω_1_, …, ω_*n*_} such that for every ω ∈ Ω, there exists ω_*r*_ ∈ Ω for which ||ω−ω_*r*_||_∞_ < *ε*. We can consider now the real sequence (ω_1_(*x*_*i*_))_*i*∈ℕ_, which is bounded since $\omega_{1}\in {\mathbb{R}}_{b}^{X}$. From Bolzano-Weierstrass Theorem, it follows that we can extract a convergent subsequence (ω_1_(*x*_*i*_*h*__))_*h*∈ℕ_. Again, we can extract from (ω_2_(*x*_*i*_*h*__))_*h*∈ℕ_ another convergent subsequence (ω_2_(*x*_*i*__*h*__*t*___))_*t*∈ℕ_. Repeating the process, we are able to extract a subsequence of (*x*_*i*_)_*i*∈ℕ_, that for simplicity of notation we can indicate as (*x*_*i*_*j*__)_*j*∈ℕ_, such that (ω_*k*_(*x*_*i*_*j*__))_*j*∈ℕ_ is a convergent subsequence in ℝ, and hence a Cauchy sequence in ℝ, for every *k* ∈ {1, …, *n*}. By construction, Ω_*ε*_ is finite, then we can find an index $\bar{ȷ}$ such that for any *k* ∈ {1, …, *n*}


$$\begin{array}{l} |\omega_{k}\left(x_{i_{\ell }}\right)-\omega_{k}\left(x_{i_{m}}\right)|\leq \varepsilon ,\;\text{for every }\ell ,m\geq \bar{ȷ}. \end{array}$$

Furthermore, we have that, for any ω ∈ Ω, any ω_*k*_ ∈ Ω_*ε*_, and any ℓ, *m* ∈ ℕ


$$\begin{array}{l} |\omega \left(x_{i_{\ell }}\right)-\omega \left(x_{i_{m}}\right)|\leq |\omega \left(x_{i_{\ell }}\right)-\omega_{k}\left(x_{i_{\ell }}\right)|+\\ \;|\omega_{k}\left(x_{i_{\ell }}\right)-\omega_{k}\left(x_{i_{m}}\right)|+|\omega_{k}\left(x_{i_{m}}\right)-\omega \left(x_{i_{m}}\right)|\\ \;\leq ||\omega -\omega_{k}{||}_{\infty }+|\omega_{k}\left(x_{i_{\ell }}\right)-\omega_{k}\left(x_{i_{m}}\right)|+\\ \;||\omega_{k}-\omega {||}_{\infty }. \end{array}$$

We observe that the choice of $\bar{ȷ}$ depends only on *ε* and Ω_*ε*_ not on *k*. Then, choosing a ω_*k*_ ∈ Ω_*ε*_ such that ||ω_*k*_−ω||_∞_ < *ε*, we get ||ω(*x*_*i*_ℓ__)−ω(*x*_*i*_*m*__)||_∞_ < 3*ε* for every ω ∈ Ω and every $\ell ,m\geq \bar{ȷ}$. Then,


$$\begin{array}{l} D_{X}^{\Omega }\left(x_{i_{\ell }},x_{i_{m}}\right)=\underset{\omega \in \Omega }{\sup}|\omega \left(x_{i_{\ell }}\right)-\omega \left(x_{i_{m}}\right)|<3\varepsilon \;\text{for every }\ell ,m\geq \bar{ȷ}. \end{array}$$

Then (*x*_*i*_*j*__)_*j*∈ℕ_ is a Cauchy sequence in *X*_Ω_. For Lemma 3.7 the statement holds.

**Corollary 3.9**. *If* Ω *is totally bounded and* *X*_Ω_ *is complete,* *X*_Ω_ *is compact*.

Proof: From Theorem 3.8, we have that *X*_Ω_ is totally bounded, and since by hypothesis it is also complete, it is compact.

Now, we will prove that the choice of the pseudo-metric $D_{X}^{\Omega }$ on *X* makes the functions in Ω non-expansive.

**Definition 3.10**. Two pseudo-metric spaces (*P, d*_*P*_) and (*Q, d*_*Q*_) are considered. A **non-expansive** function from (*P, d*_*P*_) to (*Q, d*_*Q*_) is a function *f*: *P* → *Q* such that *d*_*Q*_(*f*(*p*_1_), *f*(*p*_2_)) ≤ *d*_*P*_(*p*_1_, *p*_2_) for any *p*_1_, *p*_2_ ∈ *P*.

We denote as *NE*(*P, Q*) the space of all non-expansive functions from (*P, d*_*P*_) to (*Q, d*_*Q*_).

**Proposition 3.11**. Ω ⊆ *NE*(*X*_Ω_, ℝ).

Proof: For any *x*_1_, *x*_2_ ∈ *X*, we have that


$$|\omega \left(x_{1}\right)-\omega \left(x_{2}\right)|\leq \underset{\omega \in \Omega }{\sup}|\omega \left(x_{1}\right)-\omega \left(x_{2}\right)|=D_{X}^{\Omega }\left(x_{1},x_{2}\right).$$

Then, the topology on X induced by $D_{X}^{\Omega }$ naturally makes the measurements in Ω continuous. In particular, since the previous results hold for a generic $\Omega \subseteq {\mathbb{R}}_{b}^{X}$, they are also true for Φ and Φ′ in our model.

*Remark* 3.12. Assuming that (Φ, Φ′, *S*) is a perception triple. A function *φ*′ ∈ Φ′ may not be continuous from *X*_Φ_ to ℝ and a function *φ* ∈ Φ may not be continuous from $X_{\Phi^{\prime }}$ to ℝ. In other words, the topology on *X* induced by the pseudo-metric of one of the function spaces does not make the functions in the other continuous.

*Example* 3.13. Assuming *X* = ℝ, for every *a, b* ∈ ℝ the functions *φ*_*a*_:*X* → ℝ and $\varphi_{b}^{\prime }:X\to \mathbb{R}$ are defined by setting


$$\varphi_{a}(x)=\{\begin{array}{ll} 0\; & \text{if }x\geq a\\ 1\; & \text{otherwise} \end{array},\;\varphi_{b}^{\prime }(x)=\{\begin{array}{ll} 0\; & \text{if }x\leq b\\ 1\; & \text{otherwise} \end{array}.$$

Suppose Φ: = {*φ*_*a*_:*a* ≥ 0} and $\Phi^{\prime }:=\left\{\varphi_{b}^{\prime }:b\leq 0\right\}$. Consider the symmetry with respect to the y-axis, i.e., the map *s*(*x*) = −*x*. Surely, $s\in {\text{Aut}}_{\Phi ,\Phi^{\prime }}\left(X\right)$. We can observe that the function *φ*_1_ ∈ Φ is not continuous from $X_{\Phi }^{\prime }$ to ℝ; indeed $D_{X}^{\Phi^{\prime }}\left(0,2\right)=0$, but |*φ*_1_(0)−*φ*_1_(2)| = 1.

However, if Φ ⊆ Φ′, we have that the functions in Φ are also continuous on $X_{\Phi^{\prime }}$, indeed:

**Corollary 3.14**. *If* Φ ⊆ Φ′, *then* $\Phi \subseteq \text{NE}\left(X_{\Phi^{\prime }},\mathbb{R}\right)$.

Proof: By Proposition 3.11, the statement trivially holds since $\Phi \subseteq \Phi^{\prime }\subseteq \text{NE}\left(X_{\Phi^{\prime }},\mathbb{R}\right)$.

### 3.3 Pseudo-metrics on the space of operations

**Proposition 3.15**. *Every element of* ${\text{Aut}}_{\Phi ,\Phi^{\prime }}\left(X\right)$ *is non-expansive from* $X_{\Phi^{\prime }}$ *to* *X*_Φ_.

Proof: Considering a bijection $s\in {\text{Aut}}_{\Phi ,\Phi^{\prime }}\left(X\right)$ we have that


$$\begin{array}{l} D_{X}^{\Phi }\left(s\left(x_{1}\right),s\left(x_{2}\right)\right)=\underset{\varphi \in \Phi }{\sup}|\varphi s\left(x_{1}\right)-\varphi s\left(x_{2}\right)|\\ \;=\underset{\varphi \in \Phi s}{\sup}|\varphi \left(x_{1}\right)-\varphi \left(x_{2}\right)|\\ \;\leq \underset{\varphi^{\prime }\in \Phi^{\prime }}{\sup}|\varphi^{\prime }\left(x_{1}\right)-\varphi^{\prime }\left(x_{2}\right)|=D_{X}^{\Phi^{\prime }}\left(x_{1},x_{2}\right) \end{array}$$

for every *x*_1_, *x*_2_ ∈ *X*, where Φ*s* = {*φs*, *φ* ∈ Φ}. Then, $s\in \text{NE}\left(X_{\Phi^{\prime }},X_{\Phi }\right)$ and the statement is proved.

Now, we are ready to put more structure on ${\text{Aut}}_{\Phi ,\Phi^{\prime }}\left(X\right)$. Considering a set $\Omega \subseteq {\mathbb{R}}_{b}^{X}$ of bounded real-valued functions, we can endow the set Aut(*X*) with a pseudo-metric inherited from Ω


$$D_{\text{Aut}}^{\Omega }\left(s_{1},s_{2}\right):=\underset{\omega \in \Omega }{\sup}||\omega s_{1}-\omega s_{2}{||}_{\infty }$$

for any *s*_1_, *s*_2_ in Aut(*X*).

*Remark* 3.16. Analogously to what happens in Remark 3.6 for *X*, the sets Φ and Φ′ can endow Aut(*X*) with two possibly different pseudo-metrics $D_{\text{Aut}}^{\Phi }$ and $D_{\text{Aut}}^{\Phi^{\prime }}$. In particular, we can consider ${\text{Aut}}_{\Phi ,\Phi^{\prime }}\left(X\right)$ as a pseudo-metric subspace of Aut(*X*) with the induced pseudo-metrics.

*Remark* 3.17. We observe that, for any *s*_1_, *s*_2_ in Aut(*X*),


(3.3.1) $$\begin{array}{l} D_{\text{Aut}}^{\Omega }\left(s_{1},s_{2}\right):=\underset{\omega \in \Omega }{\sup}∥\omega s_{1}-\omega s_{2}{∥}_{\infty }\\ \;=\underset{x\in X}{\sup}\underset{\omega \in \Omega }{\sup}|\omega \left(s_{1}\left(x\right)\right)-\omega \left(s_{2}\left(x\right)\right)|\\ \;=\underset{x\in X}{\sup}D_{X}^{\Omega }\left(s_{1}\left(x\right),s_{2}\left(x\right)\right). \end{array}$$

In other words, the pseudo-metric $D_{\text{Aut}}^{\Omega }$, which is based on the action of the elements of Aut(*X*) on the set Ω, is exactly the usual uniform pseudo-metric on *X*_Ω_.

### 3.4 The space of operations

Since we are only interested in transformations of functions in Φ, it would be natural to just endow $\text{Au}{\text{t}}_{\Phi ,\Phi^{\prime }}\left(X\right)$ with the pseudo-metric $D_{\text{Aut}}^{\Phi }$. However, it is sometimes necessary to consider the pseudo-metric $D_{\text{Aut}}^{\Phi^{\prime }}$ in order to guarantee the continuity of the composition of elements in $\text{Au}{\text{t}}_{\Phi ,\Phi^{\prime }}\left(X\right)$, whenever it is admissible. Considering two elements *s, t* in ${\text{Aut}}_{\Phi ,\Phi^{\prime }}\left(X\right)$ such that *st* is still an element of ${\text{Aut}}_{\Phi ,\Phi^{\prime }}\left(X\right)$, i.e., for every function *φ* ∈ Φ, we have that *φst* ∈ Φ′. Then, for any *φ* ∈ Φ we have that


$$\begin{array}{l} \varphi^{\prime }:=\varphi s\in \Phi s\subseteq \Phi^{\prime },\;\varphi^{\prime }t\in \Phi^{\prime }. \end{array}$$

Therefore, *t* is also an element of ${\text{Aut}}_{\Phi s,\Phi^{\prime }}\left(X\right)$. By definition, Φ*s* is contained in Φ′ for every $s\in {\text{Aut}}_{\Phi ,\Phi^{\prime }}\left(X\right)$, and this justifies the choice of considering in ${\text{Aut}}_{\Phi ,\Phi^{\prime }}\left(X\right)$ also the pseudo-metric $D_{\text{Aut}}^{\Phi^{\prime }}$. We have shown, in particular, that if *s, t* are elements of ${\text{Aut}}_{\Phi ,\Phi^{\prime }}\left(X\right)$ such that *st* is still an element of ${\text{Aut}}_{\Phi ,\Phi^{\prime }}\left(X\right)$, *t* is an element of ${\text{Aut}}_{\Phi s,\Phi^{\prime }}\left(X\right)$, which is an implication of the following proposition:

**Proposition 3.18**. *Let* $s,t\in {\text{Aut}}_{\Phi ,\Phi^{\prime }}\left(X\right)$. *Then,* $st\in {\text{Aut}}_{\Phi ,\Phi^{\prime }}\left(X\right)$ *if* $t\in {\text{Aut}}_{\Phi s,\Phi^{\prime }}\left(X\right)$.

Proof: If the composition *st* belongs to ${\text{Aut}}_{\Phi ,\Phi^{\prime }}\left(X\right)$, we have already proven that $t\in {\text{Aut}}_{\Phi s,\Phi^{\prime }}\left(X\right)$. On the other hand, if $t\in {\text{Aut}}_{\Phi s,\Phi^{\prime }}\left(X\right)$, we have that $\bar{\varphi }t\in \Phi^{\prime }$ for every $\bar{\varphi }\in \Phi s$. Since *φ*(*st*) = (*φs*)*t*, it follows that *φ*(*st*) ∈ Φ′ for every *φ* ∈ Φ. Therefore, $st\in {\text{Aut}}_{\Phi ,\Phi^{\prime }}\left(X\right)$ and the statement is proved.

*Remark* 3.19. Let $t\in {\text{Aut}}_{\Phi ,\Phi^{\prime }}\left(X\right)$. We can observe that if *s* ∈ Aut_Φ_(*X*), Φ*s* ⊆ Φ and $st\in {\text{Aut}}_{\Phi ,\Phi^{\prime }}\left(X\right)$.

**Lemma 3.20**. *Consider* *r, s, t* ∈ Aut(*X*). *For any* $\Omega \subseteq {\mathbb{R}}_{b}^{X}$, *it holds that*


$$D_{\text{Aut}}^{\Omega }\left(rt,st\right)=D_{\text{Aut}}^{\Omega }\left(r,s\right).$$

Proof: Since $R_{t}$ preserves the distances, we have that:


$$\begin{array}{l} D_{\text{Aut}}^{\Omega }\left(rt,st\right):=\underset{\omega \in \Omega }{\sup}||\omega rt-\omega st{||}_{\infty }\\ \;=\underset{\omega \in \Omega }{\sup}||\omega r-\omega s{||}_{\infty }\\ \;=D_{\text{Aut}}^{\Omega }\left(r,s\right). \end{array}$$

**Lemma 3.21**. *Consider* *r, s* ∈ Aut(*X*) *and* $t\in {\text{Aut}}_{\Phi ,\Phi^{\prime }}\left(X\right)$. *It holds that*


$$D_{\text{Aut}}^{\Phi }\left(tr,ts\right)\leq D_{\text{Aut}}^{\Phi^{\prime }}\left(r,s\right).$$

Proof: Since Φ*t* ⊆ Φ′, we have that:


$$\begin{array}{l} D_{\text{Aut}}^{\Phi }\left(tr,ts\right)=\underset{\varphi \in \Phi }{\sup}||\varphi tr-\varphi ts{||}_{\infty }\\ \;=\underset{\varphi^{\prime }\in \Phi t}{\sup}||\varphi^{\prime }r-\varphi^{\prime }s{||}_{\infty }\\ \;\leq \underset{\varphi^{\prime }\in \Phi^{\prime }}{\sup}||\varphi^{\prime }r-\varphi^{\prime }s{||}_{\infty }\\ \;=D_{\text{Aut}}^{\Phi^{\prime }}\left(r,s\right). \end{array}$$

Let Π be the set of all pairs (*s, t*) such that $s,t,st\in {\text{Aut}}_{\Phi ,\Phi^{\prime }}\left(X\right)$. We endow Π with the pseudo-metric


$$D_{\Pi }\left(\left(s_{1},t_{1}\right),\left(s_{2},t_{2}\right)\right):=D_{\text{Aut}}^{\Phi }\left(s_{1},s_{2}\right)+D_{\text{Aut}}^{\Phi^{\prime }}\left(t_{1},t_{2}\right)$$

and the corresponding topology.

**Proposition 3.22**. *The function* $◦:\Pi \to \left({\text{Aut}}_{\Phi ,\Phi^{\prime }}\left(X\right),D_{\text{Aut}}^{\Phi }\right)$ *that maps* (*s, t*) *to st* *is non-expansive and hence continuous.*

Proof: Consider two elements (*s*_1_, *t*_1_), (*s*_2_, *t*_2_) of Π. By Lemma 3.20 and Lemma 3.21,


$$\begin{array}{l} D_{\text{Aut}}^{\Phi }\left(s_{1}t_{1},s_{2}t_{2}\right)\leq D_{\text{Aut}}^{\Phi }\left(s_{1}t_{1},s_{2}t_{1}\right)+D_{\text{Aut}}^{\Phi }\left(s_{2}t_{1},s_{2}t_{2}\right)\\ \;\leq D_{\text{Aut}}^{\Phi }\left(s_{1},s_{2}\right)+D_{\text{Aut}}^{\Phi^{\prime }}\left(t_{1},t_{2}\right)\\ \;=D_{\Pi }\left(\left(s_{1},t_{1}\right),\left(s_{2},t_{2}\right)\right). \end{array}$$

Therefore, the statement is proved.

Let Υ be the set of all *s* with $s,s^{-1}\in {\text{Aut}}_{\Phi ,\Phi^{\prime }}\left(X\right)$.

**Proposition 3.23**. *The function* ${\left(\cdot \right)}^{-1}:\left(\Upsilon ,D_{\text{Aut}}^{\Phi^{\prime }}\right)\to \left({\text{Aut}}_{\Phi ,\Phi^{\prime }}\left(X\right),D_{\text{Aut}}^{\Phi }\right)$, *that maps* *s* *to* *s*^−1^, *is non-expansive, and hence continuous*.

Proof: Consider two bijections *s*_1_, *s*_2_ ∈ Υ. Because of Lemma 3.20 and Lemma 3.21, we obtain that


$$\begin{array}{l} D_{\text{Aut}}^{\Phi }\left(s_{1}^{-1},s_{2}^{-1}\right)=D_{\text{Aut}}^{\Phi }\left(s_{1}^{-1}s_{2},s_{2}^{-1}s_{2}\right)\\ \;=D_{\text{Aut}}^{\Phi }\left(s_{1}^{-1}s_{2},\text{i}{\text{d}}_{X}\right)\\ \;=D_{\text{Aut}}^{\Phi }\left(s_{1}^{-1}s_{2},s_{1}^{-1}s_{1}\right)\\ \;\leq D_{\text{Aut}}^{\Phi^{\prime }}\left(s_{2},s_{1}\right)=D_{\text{Aut}}^{\Phi^{\prime }}\left(s_{1},s_{2}\right). \end{array}$$

We have previously defined the map


$$R:\Phi \times \text{Au}{\text{t}}_{\Phi ,\Phi^{\prime }}\left(X\right)\to \Phi^{\prime },\;\left(\varphi ,s\right)\mapsto \varphi s$$

where $R\left(\Phi ,s\right)=R_{s}\left(\Phi \right)$, for every $s\in {\text{Aut}}_{\Phi ,\Phi^{\prime }}\left(X\right)$.

**Proposition 3.24**. *The function* R *is continuous by choosing the pseudo-metric* $D_{\text{Aut}}^{\Phi }$ *on* ${\text{Aut}}_{\Phi ,\Phi^{\prime }}\left(X\right)$.

Proof: We have that


$$\begin{array}{l} ∥R\left(\varphi ,t\right)-R\left(\bar{\varphi },s\right){∥}_{\infty }=∥\varphi t-\bar{\varphi }s{∥}_{\infty }\\ \;\leq ||\varphi t-\varphi s{||}_{\infty }+||\varphi s-\bar{\varphi }s{||}_{\infty }\\ \;=||\varphi t-\varphi s{||}_{\infty }+||\varphi -\bar{\varphi }{||}_{\infty }\\ \;\leq D_{\text{Aut}}^{\Phi }\left(t,s\right)+||\varphi -\bar{\varphi }{||}_{\infty } \end{array}$$

for any $\varphi ,\bar{\varphi }\in \Phi$ and any $t,s\in {\text{Aut}}_{\Phi ,\Phi^{\prime }}\left(X\right)$. This proves that R is continuous.

Now, we can give a result about the compactness of $\left({\text{Aut}}_{\Phi ,\Phi^{\prime }}\left(X\right),D_{\text{Aut}}^{\Phi }\right)$ under suitable assumptions.

**Proposition 3.25**. *If* Φ *and* Φ′ *are totally bounded*, $\left({\text{Aut}}_{\Phi ,\Phi^{\prime }}\left(X\right),D_{\text{Aut}}^{\Phi }\right)$ *is totally bounded*.

Proof: Consider a sequence (*s*_*i*_)_*i*∈ℕ_ in ${\text{Aut}}_{\Phi ,\Phi^{\prime }}\left(X\right)$ and a real number *ε* > 0. Since Φ is totally bounded, we can find a finite subset Φ_*ε*_ = {*φ*_1_, …, *φ*_*n*_} such that for every *φ* ∈ Φ, there exists *φ*_*r*_ ∈ Φ for which ||*φ*−*φ*_*r*_||_∞_ < *ε*. Now, consider the sequence (*φ*_1_*s*_*i*_)_*i*∈ℕ_ in Φ′. Since also Φ′ is totally bounded, from Lemma 3.7, it follows that we can extract a Cauchy subsequence (*φ*_1_*s*_*i*_*h*__)_*h*∈ℕ_. Again, we can extract another Cauchy subsequence (*φ*_2_*s*_*i*__*h*__*t*___)_*t*∈ℕ_. Repeating the process for every *k* ∈ {1, …, *n*}, we are able to extract a subsequence of (*s*_*i*_)_*i*∈ℕ_, that for simplicity of notation we can indicate as (*s*_*i*_*j*__)_*j*∈ℕ_, such that (*φ*_*k*_*s*_*i*_*j*__)_*j*∈ℕ_ is a Cauchy sequence in Φ′ for every *k* ∈ {1, …, *n*}.

Since Φ_*ε*_ is finite, we can find an index $\bar{ȷ}$ such that for any *k* ∈ {1, …, *n*}


(3.4.1) $$\begin{array}{l} ||\varphi_{k}s_{i_{\ell }}-\varphi_{k}s_{i_{m}}{||}_{\infty }\leq \varepsilon ,\;\text{for every }\ell ,m\geq \bar{ȷ}. \end{array}$$

Furthermore, we have that for any *φ* ∈ Φ, any *φ*_*k*_ ∈ Φ_*ε*_, and any ℓ, *m* ∈ ℕ


$$\begin{array}{l} ||\varphi s_{i_{\ell }}-\varphi s_{i_{m}}{||}_{\infty }\leq ||\varphi s_{i_{\ell }}-\varphi_{k}s_{i_{\ell }}{||}_{\infty }+\\ \;||\varphi_{k}s_{i_{\ell }}-\varphi_{k}s_{i_{m}}{||}_{\infty }+||\varphi_{k}s_{i_{m}}-\varphi s_{i_{m}}{||}_{\infty }\\ \;=||\varphi -\varphi_{k}{||}_{\infty }+||\varphi_{k}s_{i_{\ell }}-\varphi_{k}s_{i_{m}}{||}_{\infty }+||\varphi_{k}-\varphi {||}_{\infty }. \end{array}$$

We observe that the choice of $\bar{ȷ}$ in (3.4.1) depends only on *ε* and Φ_*ε*_ not on *φ*. Then, choosing a *φ*_*k*_ ∈ Φ_*ε*_ such that ||*φ*_*k*_−*φ*||_∞_ < *ε*, we get ||*φs*_*i*_ℓ__−*φs*_*i*_*m*__||_∞_ < 3*ε* for every *φ* ∈ Φ and every $\ell ,m\geq \bar{ȷ}$. Hence, for every ℓ, *m* ∈ ℕ


$$\begin{array}{l} D_{\text{Aut}}^{\Phi }\left(s_{i_{\ell }},s_{i_{m}}\right)=\underset{\varphi \in \Phi }{\sup}||\varphi s_{i_{\ell }}-\varphi s_{i_{m}}{||}_{\infty }<3\varepsilon \end{array}$$

Therefore, (*s*_*i*_*j*__)_*j*∈ℕ_ is a Cauchy sequence in ${\text{Aut}}_{\Phi ,\Phi^{\prime }}\left(X\right)$. For Lemma 3.7, the statement holds.

** Corollary 4.26**. *Assume that* $S\subseteq {\text{Aut}}_{\Phi ,\Phi^{\prime }}\left(X\right)$. *If* Φ *and* Φ′ *are totally bounded and* $\left(S,D_{\text{Aut}}^{\Phi }\right)$ *is complete, it is also compact*.

Proof: From Proposition 3.25, we have that *S* is totally bounded, and since by hypothesis it is also complete, the statement holds.

## 4 The space of P-GENEOs

In this section, we introduce the concept of Partial Group Equivariant Non-Expansive Operator (P-GENEO). P-GENEOs allow us to transform data sets, preserving symmetries and distances and maintaining the acceptability conditions of the transformations. We will also describe some topological results about the structure of the space of P-GENEOs and some techniques used for defining new P-GENEOs in order to populate the space of P-GENEOs.

**Definition 4.1**. Let *X, Y* be sets and (Φ, Φ′, *S*), (Ψ, Ψ′, *Q*) be perception triples with domains *X* and *Y*, respectively. Consider a triple of functions (*F, F*′, *T*) with the following properties:

- *F*: Φ → Ψ, *F*′:Φ′ → Ψ′, *T*: *S* → *Q*;
- For any *s, t* ∈ *S* such that *st* ∈ *S* it holds that *T*(*st*) = *T*(*s*)*T*(*t*);
- For any *s* ∈ *S* such that *s*^−1^ ∈ *S* it holds that *T*(*s*^−1^) = *T*(*s*)^−1^;
- (*F, F*′, *T*) is *equivariant*, i.e., *F*′(*φs*) = *F*(*φ*)*T*(*s*) for every *φ* ∈ Φ, *s* ∈ *S*.

The triple (*F, F*′, *T*) is called a **perception map** or a **Partial Group Equivariant Operator (P-GEO)** from (Φ, Φ′, *S*) to (Ψ, Ψ′, *Q*).

In Remark 3.3, we observed that ${\text{id}}_{X}\in {\text{Aut}}_{\Phi ,\Phi^{\prime }}\left(X\right)$ if Φ ⊆ Φ′. Then, we can consider a perception triple (Φ, Φ′, *S*) with Φ ⊆ Φ′ and ${\text{id}}_{X}\in S\subseteq {\text{Aut}}_{\Phi ,\Phi^{\prime }}\left(X\right)$. Now, we will show how a P-GEO from this perception triple behaves.

** Lemma 4.2**. *Consider two perception triples* (Φ, Φ′, *S*) *and* (Ψ, Ψ′, *Q*) *with domains* *X* and *Y*, *respectively, and with* $\text{i}{\text{d}}_{X}\in S\subseteq {\text{Aut}}_{\Phi ,\Phi^{\prime }}\left(X\right)$. *Let* (*F, F*′, *T*) *be a P-GEO from* (Φ, Φ′, *S*) *to* (Ψ, Ψ′, *Q*). *Then*, Ψ ⊆ Ψ′ *and* $\text{i}{\text{d}}_{Y}\in Q\subseteq {\text{Aut}}_{\text{Ψ},{\text{Ψ}}^{\prime }}\left(Y\right)$.

Proof: Since (*F, F*′, *T*) is a P-GEO, by definition, we have that, for any *s, t* ∈ *S* such that *st* ∈ *S*, *T*(*st*) = *T*(*s*)*T*(*t*). Since id_*X*_ ∈ *S*, then


$$\begin{array}{l} T\left({\text{id}}_{X}\right)=T\left({\text{id}}_{X}{\text{id}}_{X}\right)=T\left({\text{id}}_{X}\right)T\left({\text{id}}_{X}\right) \end{array}$$

and hence $T\left({\text{id}}_{X}\right)={\text{id}}_{Y}\in Q\subseteq {\text{Aut}}_{\text{Ψ},{\text{Ψ}}^{\prime }}\left(X\right)$. Moreover, for Remark 3.3, we have that Ψ ⊆ Ψ′.

**Proposition 4.3**. *Consider two perception triples* (Φ, Φ′, *S*) *and* (Ψ, Ψ′, *Q*) *with domains* *X* *and* *Y*, *respectively, and with* $\text{i}{\text{d}}_{X}\in S\subseteq {\text{Aut}}_{\Phi ,\Phi^{\prime }}\left(X\right)$. *Let* (*F, F*′, *T*) *be a P-GEO from* (Φ, Φ′, *S*) *to* (Ψ, Ψ′, *Q*). *Then* $F^{\prime }{|}_{\Phi }=F$.

Proof: Since (*F, F*′, *T*) is a P-GEO, it is equivariant, and by Lemma 4.2, we have that


$$F^{\prime }\left(\varphi \right)=F^{\prime }\left(\varphi {\text{id}}_{X}\right)=F\left(\varphi \right)T\left({\text{id}}_{X}\right)=F\left(\varphi \right){\text{id}}_{Y}=F\left(\varphi \right)$$

for every *φ* ∈ Φ.

**Definition 4.4**. Assume that (Φ, Φ′, *S*) and (Ψ, Ψ′, *Q*) are perception triples. If (*F, F*′, *T*) is a perception map from (Φ, Φ′, *S*) to (Ψ, Ψ′, *Q*) and *F*, *F*′ are non-expansive, i.e.,


$$\begin{array}{c} ||F\left(\varphi_{1}\right)-F\left(\varphi_{2}\right){||}_{\infty }\leq ||\varphi_{1}-\varphi_{2}{||}_{\infty },\\ \;||F^{\prime }\left(\varphi_{1}^{\prime }\right)-F^{\prime }\left(\varphi_{2}^{\prime }\right){||}_{\infty }\leq ||\varphi_{1}^{\prime }-\varphi_{2}^{\prime }{||}_{\infty } \end{array}$$

for every *φ*_1_, *φ*_2_ ∈ Φ, $\varphi_{1}^{\prime },\varphi_{2}^{\prime }\in \Phi^{\prime }$, (*F, F*′, *T*) is called a **Partial Group Equivariant Non-Expansive Operator (P-GENEO)**.

In other words, a P-GENEO is a triple (*F, F*′, *T*) such that *F, F*′ are non-expansive and the following diagram commutes for every *s* ∈ *S*:


$$\begin{array}{ccc} \; & {ℛ}_{s} & \;\\ \Phi & \to & \Phi^{\prime }\\ F↓ & \; & ↓F^{\prime }\\ \; & {ℛ}_{T(s)} & \;\\ \Psi & \to & \Psi^{\prime } \end{array}$$

*Remark* 4.5. We can observe that a GENEO (see Bergomi et al., 2019) can be represented as a special case of P-GENEO, considering two perception triples (Φ, Φ′, *S*), (Ψ, Ψ′, *Q*) such that Φ = Φ′, Ψ = Ψ′, and the subsets containing the invariant transformations *S* and *Q* are groups (and then the map *T*: *S* → *Q* is a homomorphism). In this setting, a P-GENEO (*F, F*′, *T*) is a triple where the operators *F*, *F*′ are equal to each other (because of Proposition 4.3), and the map *T* is a homomorphism. Hence, instead of the triple, we can simply write the pair (*F, T*) that is a GENEO.

Considering two perception triples, we typically want to study the space of all P-GENEOs between them with the map *T* fixed. Therefore, when the map *T* is fixed and specified, we will simply consider pairs of operators (*F, F*′) instead of triples (*F, F*′, *T*), and we say that (*F, F*′) is a P-GENEO *associated with* or *with respect to* the map *T*. Moreover, in this case, we indicate the property of equivariance of the triple (*F, F*′, *T*) writing that the pair (*F, F*′) is *T*-*equivariant*.

**Figure 2:**
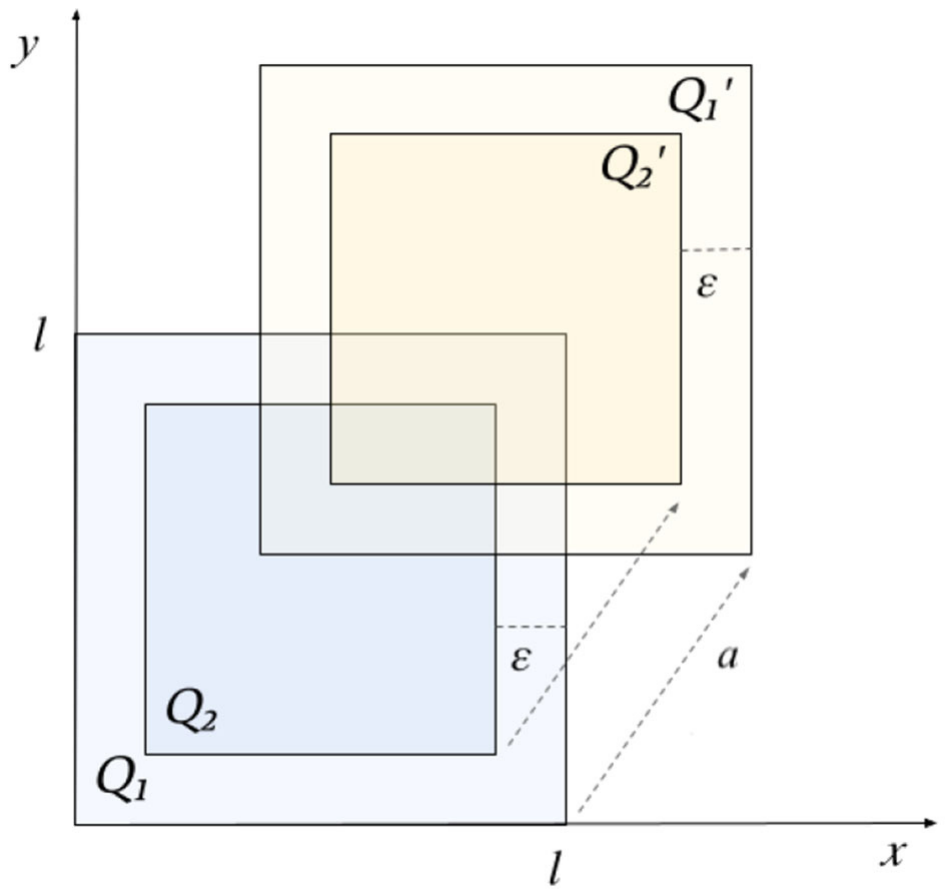
Squares used in Example 4.6.

*Example* 4.6. Let *X* = ℝ^2^. Take a real number ℓ > 0. In *X*, consider the square *Q*_1_: = [0, ℓ] × [0, ℓ] and its translation *s*_*a*_ of a vector $a=\left(a_{1},a_{2}\right)\in {\mathbb{R}}^{2}$, $Q_{1}^{\prime }:=\left[a_{1},\ell +a_{1}\right]\times \left[a_{2},\ell +a_{2}\right]$. Analogously, let us consider a real number 0 < *ε* < ℓ and two squares inside *Q*_1_ and $Q_{1}^{\prime }$, *Q*_2_: = [*ε*, ℓ−*ε*] × [*ε*, ℓ−*ε*] and $Q_{2}^{\prime }:=\left[a_{1}+\varepsilon ,\ell +a_{1}-\varepsilon \right]\times \left[a_{2}+\varepsilon ,\ell +a_{2}-\varepsilon \right]$, as shown in Figure 2.

Consider the following function spaces in ${\mathbb{R}}_{b}^{X}$:


$$\begin{array}{l} \Phi :=\left\{\varphi :X\to \mathbb{R}\;|\;\text{supp}\left(\varphi \right)\subseteq Q_{1}\right\}\\ \Phi^{\prime }:=\left\{\varphi^{\prime }:X\to \mathbb{R}\;|\;\text{supp}\left(\varphi^{\prime }\right)\subseteq Q_{1}^{\prime }\right\}\\ \text{Ψ}:=\left\{\psi :X\to \mathbb{R}\;|\;\text{supp}\left(\psi \right)\subseteq Q_{2}\right\}\\ {\text{Ψ}}^{\prime }:=\left\{\psi^{\prime }:X\to \mathbb{R}\;|\;\text{supp}\left(\psi^{\prime }\right)\subseteq Q_{2}^{\prime }\right\}. \end{array}$$

Let $S:=\left\{s_{a}^{-1}\right\}$, where *s*_*a*_ is the translation by the vector *a* = (*a*_1_, *a*_2_). The triples (Φ, Φ′, *S*) and (Ψ, Ψ′, *S*) are perception triples. This example could model the translation of two nested gray-scale images. We want to build now an operator between these images in order to obtain a transformation that commutes with the selected translation. We can consider the triple of functions (*F, F*′, *T*) defined as follows: *F*: Φ → Ψ is the operator that maintains the output of functions in Φ at points of *Q*_2_ and set them to zero outside it; analogously, *F*′:Φ′ → Ψ′ is the operator that maintains the output of functions in Φ′ at points of $Q_{2}^{\prime }$ and set them to zero outside it and *T* = id_*S*_. Therefore, the triple (*F, F*′, *T*) is a P-GENEO from (Φ, Φ′, *S*) to (Ψ, Ψ′, *S*). It turns out that the maps are non-expansive, and the equivariance holds


$$F^{\prime }\left(\varphi s_{a}^{-1}\right)=F\left(\varphi \right)T\left(s_{a}^{-1}\right)=F\left(\varphi \right)s_{a}^{-1}$$

for any *φ* ∈ Φ. From the point of view of application, we are considering two square images and their translations, and we apply an operator that “cuts” the images, taking into account only the part of the image that interests the observer. This example justifies the definition of P-GENEO as a triple of operators (*F, F*′, *T*), without requiring *F* and *F*′ to be equal in the possibly non-empty intersection of their domains. In fact, if *φ* is a function contained in Φ ∩ Φ′, its image *via* *F* and *F*′ may be different.

### 4.1 Methods to construct P-GENEOs

Starting from a finite number of P-GENEOs, we will illustrate some methods to construct new P-GENEOs. First of all, the composition of two P-GENEOs is still a P-GENEO.

**Proposition 4.7**. *Given two composable P-GENEOs,* $\left(F_{1},F_{1}^{\prime },T_{1}\right):\left(\Phi ,\Phi^{\prime },S\right)\to \left(\text{Ψ},{\text{Ψ}}^{\prime },Q\right)$ *and* $\left(F_{2},F_{2}^{\prime },T_{2}\right):\left(\text{Ψ},{\text{Ψ}}^{\prime },Q\right)\to \left(\Omega ,\Omega^{\prime },K\right)$, *their composition defined as*


$$\left(F,F^{\prime },T\right):=\left(F_{2}◦F_{1},F_{2}^{\prime }◦F_{1}^{\prime },T_{2}◦T_{1}\right):\left(\Phi ,\Phi^{\prime },S\right)\to \left(\Omega ,\Omega^{\prime },K\right)$$

*is a P-GENEO*.

Proof: First, one could easily check that the map *T* = *T*_2_ ◦ *T*_1_ respects the second and the third property of Definition 4.1. Therefore, it remains to verify that *F*(Φ) ⊆ Ω, *F*′(Φ′) ⊆ Ω′ and the properties of equivariance and non-expansiveness are maintained.

1. Since *F*_1_(Φ) ⊆ Ψ and *F*_2_(Ψ) ⊆ Ω, we have that *F*(Φ) = (*F*_2_ ◦ *F*_1_)(Φ) = *F*_2_(*F*_1_(Φ)) ⊆ *F*_2_(Ψ) ⊆ Ω. Analogously, *F*′(Φ′) ⊆ Ω′.
2. Since $\left(F_{1},F_{1}^{\prime },T_{1}\right)$ and $\left(F_{2},F_{2}^{\prime },T_{2}\right)$ are equivariant, (*F, F*′, *T*) is equivariant. Indeed, for every *φ* ∈ Φ, we have that
$$\begin{array}{l} F^{\prime }\left(\varphi s\right)=\left(F_{2}^{\prime }◦F_{1}^{\prime }\right)\left(\varphi s\right)=F_{2}^{\prime }\left(F_{1}^{\prime }\left(\varphi s\right)\right)\\ \;=F_{2}^{\prime }\left(F_{1}\left(\varphi \right)T_{1}\left(s\right)\right)=F_{2}\left(F_{1}\left(\varphi \right)\right)T_{2}\left(T_{1}\left(s\right)\right)\\ \;=\left(F_{2}◦F_{1}\right)\left(\varphi \right)\left(T_{2}◦T_{1}\right)\left(s\right)=F\left(\varphi \right)T\left(s\right). \end{array}$$
3. Since *F*_1_ and *F*_2_ are non-expansive, *F* is non-expansive; indeed for every *φ*_1_, *φ*_2_ ∈ Φ, we have that
$$\begin{array}{l} ||F\left(\varphi_{1}\right)-F\left(\varphi_{2}\right){||}_{\infty }=||\left(F_{2}◦F_{1}\right)\left(\varphi_{1}\right)-\left(F_{2}◦F_{1}\right)\left(\varphi_{2}\right){||}_{\infty }\\ \;=||F_{2}\left(F_{1}\left(\varphi_{1}\right)\right)-F_{2}\left(F_{1}\left(\varphi_{2}\right)\right){||}_{\infty }\\ \;\leq ||F_{1}\left(\varphi_{1}\right)-F_{1}\left(\varphi_{2}\right){||}_{\infty }\\ \;\leq ||\varphi_{1}-\varphi_{2}{||}_{\infty }. \end{array}$$
  Analogously, *F*′ is non-expansive.

Given a finite number of P-GENEOs with respect to the same map *T*, we illustrate a general method to construct a new operator as a combination of them. Given two sets *X* and *Y*, consider a finite set {*H*_1_, …, *H*_*n*_} of functions from $\Omega \subseteq {\mathbb{R}}_{b}^{X}$ to ${\mathbb{R}}_{b}^{Y}$ and a map $L:{\mathbb{R}}^{n}\to \mathbb{R}$, where ℝ^*n*^ is endowed with the norm $||\left(x_{1},\ldots ,x_{n}\right){||}_{\infty }:=\max_{1\leq i\leq n}|x_{i}|$. We define $L^{*}\left(H_{1},\ldots ,H_{n}\right):\Omega \to {\mathbb{R}}_{b}^{Y}$ as


$$L^{*}\left(H_{1},\ldots ,H_{n}\right)\left(\omega \right):=\left[L\left(H_{1}\left(\omega \right),\ldots ,H_{n}\left(\omega \right)\right)\right],$$

for any ω ∈ Ω, where $\left[L\left(H_{1}\left(\omega \right),\ldots ,H_{n}\left(\omega \right)\right)\right]:Y\to \mathbb{R}$ is defined by setting


$$\left[L\left(H_{1}\left(\omega \right),\ldots ,H_{n}\left(\omega \right)\right)\right]\left(y\right):=L\left(H_{1}\left(\omega \right)\left(y\right),\ldots ,H_{n}\left(\omega \right)\left(y\right)\right)$$

for any *y* ∈ *Y*. Now, consider two perception triples (Φ, Φ′, *S*) and (Ψ, Ψ′, *Q*) with domains *X* and *Y*, respectively, and a finite set of P-GENEOs $\left(F_{1},F_{1}^{\prime }\right),\ldots \left(F_{n},F_{n}^{\prime }\right)$ between them associated with the map *T*: *S* → *Q*. We can consider the functions $L^{*}\left(F_{1},\ldots ,F_{n}\right):\Phi \to {\mathbb{R}}_{b}^{Y}$ and $L^{*}\left(F_{1}^{\prime },\ldots ,F_{n}^{\prime }\right):\Phi^{\prime }\to {\mathbb{R}}_{b}^{Y}$, defined as before and state the following result.

**Proposition 4.8**. *Assume that* $L:{\mathbb{R}}^{n}\to \mathbb{R}$ is non-expansive. If $L^{*}\left(F_{1},\ldots ,F_{n}\right)\left(\Phi \right)\subseteq \text{Ψ}$ and $L^{*}\left(F_{1}^{\prime },\ldots ,F_{n}^{\prime }\right)\left(\Phi^{\prime }\right)\subseteq {\text{Ψ}}^{\prime }$, $\left(L^{*}\left(F_{1},\ldots ,F_{n}\right),L^{*}\left(F_{1}^{\prime },\ldots ,F_{n}^{\prime }\right)\right)$ *is a P-GENEO from* (Φ, Φ′, *S*) *to* (Ψ, Ψ′, *Q*) *with respect to* *T*.

Proof: By hypothesis, $L^{*}\left(F_{1},\ldots ,F_{n}\right)\left(\Phi \right)\subseteq \text{Ψ}$ and $L^{*}\left(F_{1}^{\prime },\ldots ,F_{n}^{\prime }\right)\left(\Phi^{\prime }\right)\subseteq {\text{Ψ}}^{\prime }$, so we just need to verify the properties of equivariance and non-expansiveness.

1. Since $\left(F_{1},F_{1}^{\prime }\right),\ldots ,\left(F_{n},F_{n}^{\prime }\right)$ are *T*-equivariant, for any *φ* ∈ Φ and any *s* ∈ *S*, we have that:
$$\begin{array}{l} L^{*}\left(F_{1}^{\prime },\ldots ,F_{n}^{\prime }\right)\left(\varphi s\right)=\left[L\left(F_{1}^{\prime }\left(\varphi s\right),\ldots ,F_{n}^{\prime }\left(\varphi s\right)\right)\right]\\ \;=\left[L\left(F_{1}\left(\varphi \right)T\left(s\right),\ldots ,F_{n}\left(\varphi \right)T\left(s\right)\right)\right]\\ \;=\left[L\left(F_{1}\left(\varphi \right),\ldots ,F_{n}\left(\varphi \right)\right)\right]T\left(s\right)\\ \;=L^{*}\left(F_{1},\ldots ,F_{n}\right)\left(\varphi \right)T\left(s\right). \end{array}$$
  Therefore, $\left(L^{*}\left(F_{1},\ldots ,F_{n}\right),L^{*}\left(F_{1}^{\prime },\ldots ,F_{n}^{\prime }\right)\right)$ is *T*-equivariant.
2. Since *F*_1_, …, *F*_*n*_ and L are non-expansive, for any *φ*_1_, *φ*_2_ ∈ Φ, we have that:
$$\begin{array}{l} ||L^{*}\left(F_{1},\ldots ,F_{n}\right)\left(\varphi_{1}\right)-L^{*}\left(F_{1},\ldots ,F_{n}\right)\left(\varphi_{2}\right){||}_{\infty }\\ =\underset{y\in Y}{\max}|\left[L\left(F_{1}\left(\varphi_{1}\right),\ldots ,F_{n}\left(\varphi_{1}\right)\right)\right]\left(y\right)\\ \;-\left[L\left(F_{1}\left(\varphi_{2}\right),\ldots ,F_{n}\left(\varphi_{2}\right)\right)\right]\left(y\right)|\\ =\underset{y\in Y}{\max}|L\left(F_{1}\left(\varphi_{1}\right)\left(y\right),\ldots ,F_{n}\left(\varphi_{1}\right)\left(y\right)\right)\\ \;-L\left(F_{1}\left(\varphi_{2}\right)\left(y\right),\ldots ,F_{n}\left(\varphi_{2}\right)\left(y\right)\right)|\\ \leq \underset{y\in Y}{\max}||\left(F_{1}\left(\varphi_{1}\right)\left(y\right)-F_{1}\left(\varphi_{2}\right)\left(y\right),\ldots ,F_{n}\left(\varphi_{1}\right)\left(y\right)-F_{n}\left(\varphi_{2}\right)\left(y\right)\right){||}_{\infty }\\ =\underset{y\in Y}{\max}\underset{1\leq i\leq n}{\max}|F_{i}\left(\varphi_{1}\right)\left(y\right)-F_{i}\left(\varphi_{2}\right)\left(y\right)|\\ =\underset{1\leq i\leq n}{\max}||F_{i}\left(\varphi_{1}\right)-F_{i}\left(\varphi_{2}\right){||}_{\infty }\\ \leq ||\varphi_{1}-\varphi_{2}{||}_{\infty }. \end{array}$$
  Hence, $L^{*}\left(F_{1},\ldots ,F_{n}\right)$ is non-expansive. Analogously, since $F_{1}^{\prime },\ldots ,F_{n}^{\prime }$ and L are non-expansive, $L^{*}\left(F_{1}^{\prime },\ldots ,F_{n}^{\prime }\right)$ is non-expansive.

Therefore, $\left(L^{*}\left(F_{1},\ldots ,F_{n}\right),L^{*}\left(F_{1}^{\prime },\ldots ,F_{n}^{\prime }\right)\right)$ is a P-GENEO from (Φ, Φ′, *S*) to (Ψ, Ψ′, *Q*) with respect to *T*.

*Remark 4.9*. The above result describes a general method to build new P-GENEOs, starting from a finite number of known P-GENEOs *via* non-expansive maps. Some examples of such non-expansive maps are the maximum function, the power mean, and the convex combination (for further details, see Frosini and Quercioli, 2017; Quercioli, 2021a,b).

### 4.2 Compactness and convexity of the space of P-GENEOs

Given two perception triples, under some assumptions on the data sets, it is possible to show two useful features in applications: compactness and convexity. These two properties guarantee, on the one hand, that the space of P-GENEOs can be approximated by a finite subset of them, and, on the other hand, that a convex combination of P-GENEOs is again a P-GENEO.

First, we define a metric on the space of P-GENEOs. Let *X, Y* be sets and consider two sets $\Omega \subseteq {\mathbb{R}}_{b}^{X},\Delta \subseteq {\mathbb{R}}_{b}^{Y}$, we can define the distance


$$D_{\text{NE}}^{\Omega }\left(F_{1},F_{2}\right):=\underset{\omega \in \Omega }{\sup}||F_{1}\left(\omega \right)-F_{2}\left(\omega \right){||}_{\infty }$$

for every *F*_1_, *F*_2_ ∈ **NE**(Ω, Δ).

The metric *D*_P-GENEO_ on the space $F_{T}^{all}$ of all the P-GENEOs between the perception triples (Φ, Φ′, *S*) and (Ψ, Ψ′, *Q*) associated with the map *T* is defined as


$$\begin{array}{l} D_{\text{P-GENEO}}\left(\left(F_{1},F_{1}^{\prime }\right),\left(F_{2},F_{2}^{\prime }\right)\right):=\max\left\{D_{\text{NE}}^{\Phi }\left(F_{1},F_{2}\right),D_{\text{NE}}^{\Phi^{\prime }}\left(F_{1}^{\prime },F_{2}^{\prime }\right)\right\}\\ =\max\left\{\underset{\varphi \in \Phi }{\sup}||F_{1}\left(\varphi \right)-F_{2}\left(\varphi \right){||}_{\infty },\underset{\varphi^{\prime }\in \Phi^{\prime }}{\sup}||\overset{\prime }{\underset{1}{F}}\left(\varphi^{\prime }\right)-\overset{\prime }{\underset{2}{F}}\left(\varphi^{\prime }\right){||}_{\infty }\right\} \end{array}$$

for every $\left(F_{1},F_{1}^{\prime }\right),\left(F_{2},F_{2}^{\prime }\right)\in F_{T}^{all}$.

#### 4.2.1 Compactness

Before proceeding, we need to prove that the following result holds:

**Lemma 4.10**. *If* (*P, d*_*P*_), (*Q, d*_*Q*_) *are compact metric spaces*, **NE**(*P, Q*) *is compact*.

Proof: Theorem 5 in the study by Li et al. (2012) implies that **NE**(*P, Q*) is relatively compact, since it is a equicontinuous space of maps. Hence, it will suffice to show that **NE**(*P, Q*) is closed. Considering a sequence (*F*_*i*_)_*i*∈ℕ_ in **NE**(*P, Q*) such that $\lim_{i\to \infty }F_{i}=F$, we have that


$$\begin{array}{l} d_{Q}\left(F\left(p_{1}\right),F\left(p_{2}\right)\right)=\underset{i\to \infty }{\lim}d_{Q}\left(F_{i}\left(p_{1}\right),F_{i}\left(p_{2}\right)\right)\leq d_{P}\left(p_{1},p_{2}\right) \end{array}$$

for every *p*_1_, *p*_2_ ∈ *P*. Therefore, *F* ∈ **NE**(*P, Q*). It follows that **NE**(*P, Q*) is closed.

Consider two perception triples (Φ, Φ′, *S*) and (Ψ, Ψ′, *Q*), with domains *X* and *Y*, respectively, and the space $F_{T}^{all}$ of P-GENEOs between them associated with the map *T*: *S* → *Q*. The following result holds:

**Theorem 4.11**. *If* Φ, Φ′, Ψ *and* Ψ′ *are compact,* $F_{T}^{all}$ *is compact with respect to the metric* *D*_P − GENEO_.

Proof: By definition, $F_{T}^{all}\subseteq \text{NE}\left(\Phi ,\text{Ψ}\right)\times \text{NE}\left(\Phi^{\prime },{\text{Ψ}}^{\prime }\right)$. Since Φ, Φ′, Ψ and Ψ′ are compact, for Lemma 4.10, the spaces **NE**(Φ, Ψ) and **NE**(Φ′, Ψ′) are also compact, and then, by Tychonoff's Theorem, the product **NE**(Φ, Ψ) × **NE**(Φ′, Ψ′) is also compact, with respect to the product topology. Hence, to prove our statement, it suffices to show that $F_{T}^{all}$ is closed. Let us consider a sequence ${\left(\left(F_{i},F_{i}^{\prime }\right)\right)}_{i\in \mathbb{N}}$ of P-GENEOs, converging to a pair (*F, F*′) ∈ **NE**(Φ, Ψ) × **NE**(Φ′, Ψ′). Since $\left(F_{i},F_{i}^{\prime }\right)$ is *T*-equivariant for every *i* ∈ ℕ and the action of *Q* on Ψ is continuous (see Proposition 3.24), (*F, F*′) belongs to $F_{T}^{all}$. Indeed, we have that


$$\begin{array}{l} F^{\prime }\left(\varphi s\right)=\underset{i\to \infty }{\lim}F_{i}^{\prime }\left(\varphi s\right)=\underset{i\to \infty }{\lim}F_{i}\left(\varphi \right)T\left(s\right)=F\left(\varphi \right)T\left(s\right) \end{array}$$

for every *s* ∈ *S* and every *φ* ∈ Φ. Hence, $F_{T}^{all}$ is a closed subset of a compact set, and then, it is also compact.

#### 4.2.2 Convexity

Assume that Ψ, Ψ′ are convex. Let $\left(F_{1},F_{1}^{\prime }\right),\ldots ,\left(F_{n},F_{n}^{\prime }\right)\in F_{T}^{all}$ and consider an *n*-tuple $\left(a_{1},\ldots ,a_{n}\right)\in {\mathbb{R}}^{n}$ with *a*_*i*_ ≥ 0 for every *i* ∈ {1, …, *n*} and $\overset{n}{\underset{i=1}{\sum }}a_{i}=1$. We can define two operators *F*_Σ_:Φ → Ψ and $F_{\Sigma }^{\prime }:\Phi^{\prime }\to \Psi^{\prime }$ as


$$F_{\Sigma }(\varphi ):=\sum_{i=1}^{n}a_{i}F_{i}(\varphi ),\text{ and }F_{\Sigma }^{\prime }(\varphi^{\prime }):=\sum_{i=1}^{n}a_{i}F_{i}^{\prime }(\varphi^{\prime })$$

for every *φ* ∈ Φ, *φ*′ ∈ Φ′. We notice that the convexity of Ψ and Ψ′ guarantees that *F*_Σ_ and $F_{\Sigma }^{\prime }$ are well defined.

**Proposition 4.12**. $\left(F_{\Sigma },F_{\Sigma }^{\prime }\right)$ *belongs to* $F_{T}^{all}$.

Proof: By hypothesis, for every *i* ∈ {1, …, *n*}, $\left(F_{i},F_{i}^{\prime }\right)$ is a perception map, and then:


$$\begin{array}{l} F_{\Sigma }^{\prime }(\varphi s)=\sum_{i=1}^{n}a_{i}{F^{\prime }}_{i}(\varphi s)=\sum_{i=1}^{n}a_{i}(F_{i}(\varphi )T(s))\\ \;=(\sum_{i=1}^{n}a_{i}F_{i}(\varphi ))T(s)\\ \;=F_{\Sigma }(\varphi )T(s) \end{array}$$

for every *φ* ∈ Φ and every *s* ∈ *S*. Furthermore, since for every *i* ∈ {1, …, *n*}, *F*_*i*_(Φ) ⊆ Ψ and Ψ are convex, also *F*_Σ_(Φ) ⊆ Ψ. Analogously, the convexity of Ψ′ implies that $F_{\Sigma }^{\prime }(\Phi^{\prime })\subseteq \Psi^{\prime }$. Therefore $\left(F_{\Sigma },F_{\Sigma }^{\prime }\right)$ is a P-GEO. It remains to show the non-expansiveness of *F*_Σ_ and $F_{\Sigma }^{\prime }$. Since *F*_*i*_ is non-expansive for any *i*, for every *φ*_1_, *φ*_2_ ∈ Φ, we have that


$$\begin{array}{l} ||F_{\Sigma }\left(\varphi_{1}\right)-F_{\Sigma }\left(\varphi_{2}\right){||}_{\infty }=||\overset{n}{\underset{i=1}{\sum }}a_{i}F_{i}\left(\varphi_{1}\right)-\overset{n}{\underset{i=1}{\sum }}a_{i}F_{i}\left(\varphi_{2}\right){||}_{\infty }\\ \;=||\overset{n}{\underset{i=1}{\sum }}a_{i}\left(F_{i}\left(\varphi_{1}\right)-F_{i}\left(\varphi_{2}\right)\right){||}_{\infty }\\ \;\leq \overset{n}{\underset{i=1}{\sum }}|a_{i}|||F_{i}\left(\varphi_{1}\right)-F_{i}\left(\varphi_{2}\right){||}_{\infty }\\ \;\leq \overset{n}{\underset{i=1}{\sum }}|a_{i}|||\varphi_{1}-\varphi_{2}{||}_{\infty }=||\varphi_{1}-\varphi_{2}{||}_{\infty }. \end{array}$$

Analogously, since every $F_{i}^{\prime }$ is non-expansive, for every $\varphi_{1}^{\prime },\varphi_{2}^{\prime }\in \Phi^{\prime }$, we have that


$$\|F_{\Sigma }^{\prime }(\varphi_{1}^{\prime })-F_{\Sigma^{\prime }}(\varphi_{2}^{\prime }){\|}_{\infty }\leq \sum_{i=1}^{n}|a_{i}|\|\varphi_{1}^{\prime }-\varphi_{2}^{\prime }{\|}_{\infty }=\|\varphi_{1}^{\prime }-\varphi_{2}^{\prime }{\|}_{\infty }.$$

Therefore, we have proven that $\left(F_{\Sigma },F_{\Sigma }^{\prime }\right)$ is a P-GEO with *F*_Σ_ and $F_{\Sigma }^{\prime }$ non-expansive. Hence it is a P-GENEO.

Then, the following result holds:

**Corollary 4.13**. *If* Ψ, Ψ′ *are convex, the set* $F_{T}^{all}$ *is convex*.

Proof: It is sufficient to apply Proposition 4.12 for *n* = 2 by setting *a*_1_ = *t*, *a*_2_ = 1−*t* for 0 ≤ *t* ≤ 1.

## 5 P-GENEOs in applications

**Figure 3:**
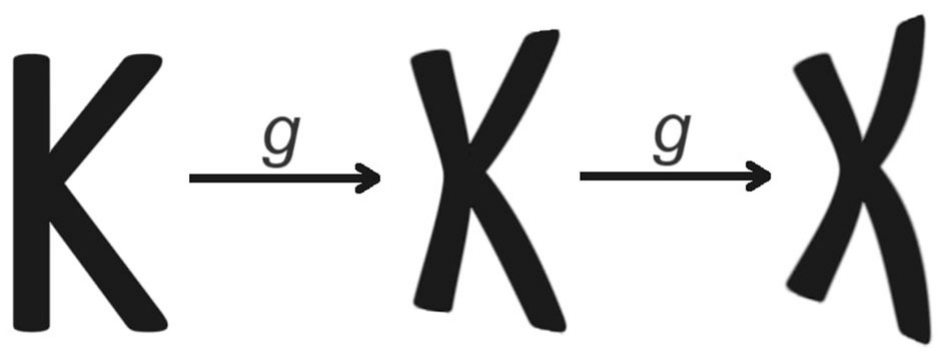
Applying a “shape-preserving” homeomorphism twice can change a letter *k* into a letter *x*.

The importance of equivariance with respect to a group is becoming clear and widespread in many machine learning applications used for drug design, traffic forecasting, object recognition, and detection (see, e.g., Bronstein et al., 2021; Gerken et al., 2023). In some situations, however, requiring equivariance with respect to a whole group could even become an obstacle in the correct learning process of an equivariant neural network. In the following, we describe a possible application to optical character recognition (OCR), in which partial equivariance might be better suited than equivariance. Consider a planar transformation that deforms characters. One may notice that if such transformation is performed too many times, the letter may lose or change its meaning, as shown in Figure 3. Another example is given by a reparameterization of the domain of a sound message. While a limited contraction or dilation of the domain can preserve the meaning attributed to the sound, an iterated application of the same transformation can radically change the perceived message.

Furthermore, experiments performed in the study by Weiler and Cesa (2019) have shown that tuning the level of equivariance in each layer of a neural network may increase the performance of the model. This tuning is, however, performed manually. The successive step, conducted in the study by Romero and Lohit (2022), is to learn the level of equivariance of each layer directly from data, possibly restricting to certain subsets whenever the full equivariance prevents a good classification performance. The authors of Romero and Lohit (2022) test their result on MNIST. In applications of this type, the use of P-GENEOs could allow partial equivariance to be framed within a precise mathematical model.

## 6 Conclusion

In this article, we proposed a generalization of some known results in the theory of GENEOs to a new mathematical framework, where the collection of all symmetries is represented by a subset of a group of transformations. We introduced P-GENEOs and showed that they are a generalization of GENEOs. We defined pseudo-metrics on the space of measurements and on the space of P-GENEOs and studied their induced topological structures. Under the assumption that the function spaces are compact and convex, we showed compactness and convexity of the space of P-GENEOs. In particular, compactness guarantees that any operator can be approximated by a finite number of operators belonging to the same space, while convexity allows us to build new P-GENEOs by taking convex combinations of P-GENEOs. Compactness and convexity together ensure that every strictly convex loss function on the space of P-GENEOs admits a unique global minimum. Given a collection of P-GENEOs, we presented a general method to construct new P-GENEOs as combinations of the initial ones.

## Data availability statement

The original contributions presented in the study are included in the article/supplementary material, further inquiries can be directed to the corresponding author.

## Author contributions

LF: Writing – original draft. PF: Writing – original draft, Writing – review & editing. NQ: Writing – original draft, Writing – review & editing. FT: Writing – original draft, Writing – review & editing.
